# Tower Hamlets Community Learning Disability Service: Sodium Valproate Audit for Male Patients With Learning Disabilities

**DOI:** 10.1192/bjo.2025.10686

**Published:** 2025-06-20

**Authors:** Huan Tan, Sehrish Ali, David Prior, Kainat Khan, Nicole Eady

**Affiliations:** East London NHS Foundation Trust, London, United Kingdom

## Abstract

**Aims:** People with learning disabilities are at a higher risk of developing epilepsy and bipolar disorder. For decades, sodium valproate has been used to treat these conditions. However, recent evidence suggests an increased risk of testicular toxicity in men and neurodevelopmental disorders in children born to men who were treated with valproate in the three months prior to conception. Sodium valproate is not recommended for male patients under 55 years of age unless no other effective or tolerated treatment is available.

Aims were:

To compare our prescribing practices with the latest guidelines.

To review the indication for sodium valproate in male patients with learning disabilities.

To explain the potential risks of infertility and testicular toxicity.

**Methods:** We conducted a cross-sectional study within our service, collecting data on all male patients currently taking sodium valproate, focusing on their age, diagnosis, and dosage. We then contacted these patients to complete the ‘Risk Acknowledgement Form’ which involves three steps:

1. Documentation of the prescribing decision.

2. Explanation of the risks to the patient.

3. Countersignature by both the patient and clinician.

**Results:** A total of 25 male patients are taking sodium valproate under our service. Of these, 16 patients are aged under 40, 6 are aged 41–50, and 3 are over 50. Ten patients have bipolar disorder, 2 have schizoaffective disorder, and 12 have epilepsy, with one patient diagnosed with both epilepsy and bipolar disorder.

Regarding dosage, 5 patients are taking less than 1000 mg per day, 18 patients are taking between 1000–2000 mg per day, and 2 are taking more than 2000 mg per day. Of the 25 patients, 10 have completed the safety questionnaires. Additionally, 11 patients receive their sodium valproate from other services, such as GPs or neurologists, while 4 patients remain pending due to reasons such as inability to contact or lack of capacity.

**Conclusion:** This audit highlights the ongoing use of sodium valproate in male patients in our service. Despite concerns about its risks – particularly testicular toxicity and potential impacts on fertility – sodium valproate remains one of the most effective treatments available.

The results indicate that a small number of patients are receiving doses exceeding the recommended BNF thresholds due to clinical complexity.

Moving forward, further efforts should be made to reduce sodium valproate dosages and switch to alternative mood stabilizers when possible. Additionally, services should prioritize enhancing communication and documentation of potential risks while continuing to monitor and mitigate any adverse effects.

